# Secretory production of an FAD cofactor-containing cytosolic enzyme (sorbitol–xylitol oxidase from *Streptomyces* coelicolor) using the twin-arginine translocation (Tat) pathway of *Corynebacterium glutamicum*

**DOI:** 10.1111/1751-7915.12005

**Published:** 2012-11-20

**Authors:** Sandra Scheele, Dan Oertel, Johannes Bongaerts, Stefan Evers, Hendrik Hellmuth, Karl-Heinz Maurer, Michael Bott, Roland Freudl

**Affiliations:** 1 Institut für Bio- und Geowissenschaften 1, Biotechnologie, Forschungszentrum Jülich GmbHD-52425, Jülich, Germany; 2 International R&D/Technology Laundry and Home Care, Henkel AG & Co. KGaAD-40191, Düsseldorf, Germany

## Abstract

Carbohydrate oxidases are biotechnologically interesting enzymes that require a tightly or covalently bound cofactor for activity. Using the industrial workhorse *Corynebacterium glutamicum* as the expression host, successful secretion of a normally cytosolic FAD cofactor-containing sorbitol–xylitol oxidase from *Streptomyces coelicolor* was achieved by using the twin-arginine translocation (Tat) protein export machinery for protein translocation across the cytoplasmic membrane. Our results demonstrate for the first time that, also for cofactor-containing proteins, a secretory production strategy is a feasible and promising alternative to conventional intracellular expression strategies.

The secretory expression of recombinant proteins can offer significant process advantages over cytosolic production strategies, since secretion into the growth medium greatly facilitates downstream processing and therefore can significantly reduce the costs of producing a desired target protein (Quax, 1997). And, in fact, the enormous secretion capacity of certain Gram-positive bacteria (e.g. various *Bacillus* species) has been used since many years in industry for the production of mainly host-derived secretory proteins such as proteases and amylases, resulting in amounts of more than 20 g l^−1^ culture medium (Harwood and Cranenburg, 2008). In contrast, attempts to use *Bacillus* species for the secretory production of heterologous proteins have often failed or led to disappointing results, a fact that, among other reasons, could in many cases be attributed to the presence of multiple cell wall-associated and secreted proteases that rapidly degraded the heterologous target proteins (Li *et al*., 2004; Sarvas *et al*., 2004; Westers *et al*., 2011). Therefore, an increasing need exists to explore alternative host systems with respect to their ability to express and secrete problematic and/or complex heterologous proteins of biotechnological interest.

So far, the Gram-positive bacterium *Corynebacterium glutamicum* has been used in industry mainly for the production of amino acids and other low-molecular weight compounds (Leuchtenberger *et al*., 2005; Becker and Wittmann, 2011; Litsanov *et al*., 2012). However, various recent reports have indicated that *C. glutamicum* might likewise possess a great potential as an alternative host system for the secretory expression of foreign proteins. *Corynebacterium glutamicum* belongs to a class of diderm Gram-positive bacteria that, besides the cytoplasmic membrane, possess an additional mycolic acid-containing outer membrane-like structure that acts as an extremely efficient permeability barrier for hydrophilic compounds (Hoffmann *et al*., 2008; Zuber *et al*., 2008). Despite this fact, an efficient secretion of various heterologous proteins into the growth medium of this microorganism has been observed (e.g. Billman-Jacobe *et al*., 1995; Meissner *et al*., 2007; Kikuchi *et al*., 2009; Tateno *et al*., 2009; Tsuchidate *et al*., 2011).

In bacteria, two major export pathways exist for the transport of proteins across the cytoplasmic membrane that fundamentally differ with respect to the folding status of their respective substrate proteins during the actual translocation step. The general secretion (Sec) system transports its substrates in a more or less unfolded state and folding takes places on the trans side of the membrane after the actual transport event (Yuan *et al*., 2010; du Plessis *et al*., 2011). In contrast, the alternative twin-arginine translocation (Tat) system translocates its substrates in a fully folded form and therefore provides an attractive alternative for the secretory production of proteins that cannot be produced in a functional form via the Sec route (Brüser, 2007). Carbohydrate oxidases are biotechnologically interesting enzymes (van Hellemond *et al*., 2006) that are excluded from Sec-dependent secretion since they depend on a tightly or covalently bound cofactor for their activity and, for this reason, require that their folding and cofactor insertion has to take place in the cytosol. Because *C. glutamicum* has shown to be an excellent host for the Tat-dependent secretion of the cofactor-less model protein GFP (Meissner *et al*., 2007; Teramoto *et al*., 2011), we now asked whether it is likewise possible to secrete a cofactor-containing enzyme into the supernatant of *C. glutamicum* using the same protein export route.

As a model protein, we chose the sorbitol–xylitol oxidase (SoXy) from *Streptomyces coelicolor*, a normally cytosolic enzyme that possesses a covalently bound FAD molecule as cofactor (Heuts *et al*., 2007; Forneris *et al*., 2008). FAD is incorporated into the apoprotein in a post-translational and self-catalytic process that only occurs if the polypeptide chain has adopted a correctly folded structure (Heuts *et al*., 2007; 2009). To direct SoXy into the Tat export pathway of *C. glutamicum*, we constructed a gene encoding a TorA–SoXy hybrid precursor in which SoXy is fused to the strictly Tat-specific signal peptide of the periplasmic *Escherichia coli* Tat substrate trimethylamine N-oxide reductase (TorA) (Fig. 1) which, in our previous study, has been proven to be a functional and strictly Tat-specific signal peptide also in *C. glutamicum* (Meissner *et al*., 2007). The corresponding *torA*–*soxy* gene was cloned into the expression vector pEKEx2 (Eikmanns *et al*., 1991) under the control of an IPTG-inducible P_tac_ promotor. After transformation of the resulting plasmid pTorA–SoXy into the *C. glutamicum* ATCC13032 wild-type strain, two independent colonies of the resulting recombinant *C. glutamicum* (pTorA–SoXy) strain and, as a control, a colony of a strain that contained the empty expression vector without insert [*C. glutamicum* (pEKEx2)] were grown in CGXII medium (Keilhauer *et al*., 1993) at 30°C for 16 h in the presence of 1 mM IPTG. Subsequently, the proteins present in the culture supernatants were analysed by SDS-PAGE followed by staining with Coomassie blue. As shown in Fig. 2, in the supernatants of the pTorA–SoXy-containing cells (lanes 3 and 4), a prominent protein band of approximately 44 kDa can be detected, the size of which is very similar to the calculated molecular mass (44.4 kDa) of SoXy. Since this band is completely lacking in the supernatant of the control strain (lane 2), this strongly suggests that this band corresponds to SoXy that has been secreted into the culture supernatant of *C. glutamicum* (pTorA–SoXy). And, in fact, this suggestion was subsequently confirmed in a direct way by MALDI-TOF mass spectrometry after extraction of the protein out of the gel followed by tryptic digestion (Schaffer *et al*., 2001) (data not shown).

**Figure 1:**
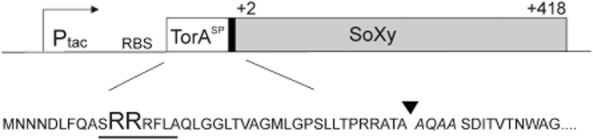
The TorA–SoXy hybrid precursor protein. Upper part: Schematic drawing of the relevant part of the pTorA–SoXy expression vector. P_tac_, IPTG-inducible *tac* promotor. RBS, ribosome binding site. To maintain the authentic TorA signal peptidase cleavage site, the first four amino acids of the mature TorA protein (black bar) were retained in the TorA–SoXy fusion protein. White bar: TorA signal peptide (TorA^SP^); grey bar: SoXy (amino acids 2–418). Lower part: Amino acid sequence of the signal peptide and early mature region of the TorA–SoXy hybrid precursor. The twin-arginine consensus motif of the TorA signal peptide is underlined. The four amino acids derived from mature TorA are shown in italics. The signal peptidase cleavage site is indicated by an arrowhead.

**Figure 2:**
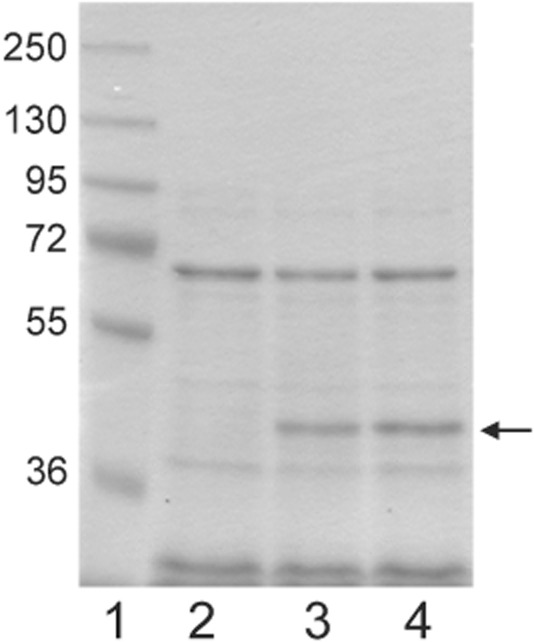
Secretion of SoXy into the growth medium of *C. glutamicum*. Cells of *C. glutamicum* ATCC13032 containing the empty vector pEKEx2 and two independently transformed colonies of *C. glutamicum* (pTorA–SoXy) were grown overnight in 5 ml of BHI medium (Difco) at 30°C. The cells were washed once with CGXII medium (Keilhauer *et al*., 1993) and inoculated to an OD_600_ of 0.5 in 5 ml of fresh CGXII medium containing 1 mM IPTG. After 16 h of further growth at 30°C, the supernatant fractions were prepared as described previously (Meissner *et al*., 2007). Samples corresponding to an equal number of cells were subjected to SDS-PAGE followed by staining with Coomassie blue. Lane 1, molecular mass marker (kDa). Lane 2, *C. glutamicum* (pEKEx2); lanes 3 and 4, *C. glutamicum* (pTorA–SoXy). The position of the secreted SoXy protein is indicated by an arrow.

Next, the supernatant of *C. glutamicum* (pTorA–SoXy) was analysed for SoXy enzyme activity by measuring the production of H_2_O_2_ that is formed during the enzymatic conversion of sorbitol to fructose (Meiattini, 1983). Six hours after induction of gene expression by 1 mM IPTG, an enzymatic activity of 10.3 ± 1.6 nmol min^−1^ ml^−1^ could be determined in the supernatant of *C. glutamicum* (pTorA–SoXy). In contrast, no such activity was found in the supernatant of the control strain *C. glutamicum* (pEKEx2). From these results we conclude that we have succeeded in the secretion of enzymatically active and therefore FAD cofactor-containing SoXy into the culture supernatant of *C. glutamicum*.

Finally, we examined whether the secretion of SoXy had in fact occurred via the Tat pathway of *C. glutamicum*. Plasmid pTorA–SoXy was used to transform *C. glutamcium* ATCC13032 wild type and a *C. glutamicum* ΔTatAC mutant strain that lacks two essential components of the Tat transport machinery and therefore does not possess a functional Tat translocase (Meissner *et al*., 2007). The corresponding cells were grown in BHI medium (Difco) at 30°C in the presence of 1 mM IPTG for 6 h. Subsequently, the proteins present in the cellular and the supernatant fractions of the corresponding cells were analysed by SDS-PAGE followed by Western blotting using SoXy-specific antibodies. As shown in Fig. 3, polypeptides corresponding to the unprocessed TorA–SoXy precursor and some minor smaller degradation products of it can be detected in the cellular fractions of both the wild-type and the ΔTatAC deletion strains (lanes 3 and 5). In the supernatant fraction of the Tat^+^ wild-type strain (lane 4), but not that of the ΔTatAC strain (lane 6), a polypeptide corresponding to mature SoXy is present, clearly showing that export of SoXy in the wild-type strain had occurred in a strictly Tat-dependent manner. Another noteworthy finding is the observation that hardly any mature SoXy protein accumulated in the cellular fraction of the Tat^+^ wild-type strain (lane 3), indicating that SoXy is, after its Tat-dependent translocation across the cytoplasmic membrane and processing by signal peptidase, rapidly transported out of the intermembrane space across the mycolic acid-containing outer membrane into the supernatant. However, the mechanism of how proteins cross this additional permeability barrier is completely unknown so far (Bitter *et al*., 2009).

**Figure 3:**
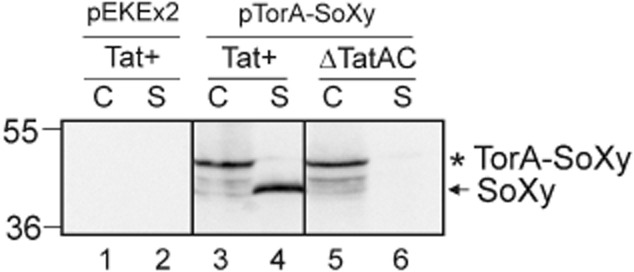
Transport of TorA–SoXy occurs in a strictly Tat-dependent manner. Plasmid pTorA–SoXy was transformed into *C. glutamcium* ATCC13032 (Tat^+^) and a *C. glutamicum* ΔTatAC mutant that lacks a functional Tat translocase (Meissner *et al*., 2007). As a control, the empty pEKEx2 expression vector was transformed into *C. glutamicum* ATCC13032 (Tat^+^). The respective strains were grown overnight in 5 ml of BHI medium (Difco) at 30°C. The cells were washed once with BHI and resuspended in 20 ml of fresh BHI medium containing 1 mM IPTG. After 6 h of further growth at 30°C, the cellular (C) and supernatant (S) fractions were prepared as described previously (Meissner *et al*., 2007). Samples of the C and S fractions were subjected to SDS-PAGE followed by immunoblotting using anti-SoXy antibodies as indicated at the top of the figure. Lanes 1 and 2: *C. glutamicum* ATCC13032 (pEKEx2); lanes 3 and 4: *C. glutamicum* ATCC13032 (pTorA–SoXy); lanes 5 and 6: *C. glutamicum* ΔTatAC (pTorA–SoXy). Asterisk: TorA–SoXy precursor; arrow: secreted SoXy protein. The positions of molecular mass markers (kDa) are indicated at the left margin of the figure.

To the best of our knowledge, our results represent the first documented example of the successful secretion of a normally cytosolic, cofactor-containing protein via the Tat pathway in an active form into the culture supernatant of a recombinant expression host. Our results clearly show that, also for this biotechnologically very interesting class of proteins, a secretory production strategy can be a promising alternative to conventional intracellular expression strategies. Besides for SoXy and other FAD-containing carbohydrate oxidases, for which various applications are perceived by industry such as the *in situ* generation of hydrogen peroxide for bleaching and disinfection performance in technical applications, their use in the food and drink industry, as well as their use in diagnostic applications and carbohydrate biosynthesis processes (Oda and Hiraga, 1998; Murooka and Yamashita, 2001; van Hellemond *et al*., 2006; Heuts *et al*., 2007), a secretory production strategy might now be an attractive option also for biotechnologically relevant enzymes that are used as biocatalysts in chemo-enzymatic syntheses and that possess cofactors other than FAD, such as pyridoxal-5′-phosphate (PLP)-dependent ω-transaminases (Mathew and Yun, 2012) or various thiamin diphosphate (TDP)-dependent enzymes (Müller *et al*., 2009).
